# Profiles of total and sn-2 fatty acid of human mature milk and their correlated factors: A cross-sectional study in China

**DOI:** 10.3389/fnut.2022.926429

**Published:** 2022-08-22

**Authors:** Mengmei Ni, Yingyao Wang, Zhirui Yang, Xuebing Xu, Hong Zhang, Yuexin Yang, Lishi Zhang, Jinyao Chen

**Affiliations:** ^1^Department of Nutrition and Food Safety, West China School of Public Health and West China Fourth Hospital, Sichuan University, Chengdu, China; ^2^Chinese Nutrition Society, Beijing, China; ^3^CNS Academy of Nutrition and Health (Beijing Zhongyinghui Nutrition and Health Research Institute) Beijing Zhongyinghui Nutrition and Health Research Institute, Beijing, China; ^4^Wilmar (Shanghai) Biotechnology Research & Development Center Co., Ltd., Shanghai, China; ^5^Chinese Center for Disease Control and Prevention, National Institute for Nutrition and Health, Beijing, China

**Keywords:** breast milk, mature milk, total fatty acid, sn-2 fatty acid, sociodemographic factors

## Abstract

Fatty acid (FA) in breast milk is beneficial to the growth and neurodevelopment of infants. However, the structure profiles of breast milk FAs and the influencing factors which are crucial for normal function have not been fully elucidated. This study aimed to characterize the profiles of total and sn-2 FAs in human mature milk based on two representative urban areas in China and explore potential sociodemographic determinants. Mothers (*n* = 70) at 40–100 d postpartum from Beijing and Danyang were recruited according to unified inclusion and exclusion criteria. Total and sn-2 FA compositions were examined by gas chromatography and quantified. Using the Spearman correlation and multiple regression model, we found that the location and maternal education level were the most conspicuous correlated factor. The milk of mothers from Beijing had higher levels of the n-6 series of long-chain polyunsaturated fatty acids (LCPUFA) (C20:2, C20:3n-6, C20:4n-6, n-6PUFA/n-3PUFA, LA/ALA, and ARA/DHA) than that of Danyang, while the opposite was observed in the n-3 series of LCPUFA (C18:3n-3 and Total n-3PUFA). Compared to the milk of mothers with a high school degree or below, those with a bachelor's degree or above had lower SFAs (C10:0, C12:0, C14:0, and Total SFA), n-3 series of LCPUFA (C18:3n-3 and Total n-3PUFA), C18:1n-9t, and higher n-6 series of LCPUFA (C18:2n-6c, C20:2, C20:4n-6, Total n-6PUFA, and n-6PUFA/n-3PUFA). Maternal age, infant gender, pre-conception body mass index (BMI), parity, delivery mode, and gestational weight gain were also associated with total FAs. However, fewer associations were found between the above factors and sn-2 FAs. This study will promote an understanding of human breast milk's lipid profile and help develop a formula more suitable for infants.

## Introduction

Human milk is the optimal food for infants during the first 6 months of life. It provides adequate nutrients and numerous bioactive ingredients such as water, carbohydrate, fat, protein, minerals, vitamins, immunoglobulin, and lactoferrin (1–3). However, the composition of human milk differs significantly between and within mothers. Human milk fatty acids (FAs) are the most variable macronutrient (4). The lipid content of human milk varies between 3 and 5% mostly (wider variation also has been reported) (5); it is the major energy source in breast milk and provides 40–50% of an infant's daily energy requirement (6–8). Triacylglycerol (TAG) is the major compound of lipid in breast milk. The property of TAGs mainly depends on the composition and specific position distribution of FAs. Studies have found that saturated fatty acid (SFA), especially esterified palmitic acid (PA; C16:0), preferentially occupies the sn-2 position in human milk (9, 10), while in formulas, PA is mainly located at the sn-1,3 positions (11, 12). A high level of PA at the sn-2 position is reported to promote the absorption of fat and calcium in infants (13), reduce insoluble calcium soap in feces (14), and aid in intestinal microbiota development of infants (15). However, the FA composition in human milk is easily influenced by maternal characteristics (dietary habits (3), lactation, and gestational age (16), duration of pregnancy (12), the stage of lactation (9, 17), and body mass index (BMI) (18, 19) and also external factors such as maternal geographic location (3, 17) and socioeconomic status (2)).

A number of studies have investigated the impact of maternal dietary habits and lactation stage on FA composition in mother's milk (3, 8, 20, 21). Nevertheless, the investigation of profiles of sn-2 FAs in human mature milk is limited, with only a few reported data for the Chinese population (10, 22, 23). Meanwhile, the exploration of influencing sociodemographic factors of sn-2 FAs is still rarely reported.

In this study, we assessed the total and sn-2 FA profiles of mature milk from mothers living in Beijing and Danyang, China. The associations between FA composition and sociodemographic factors (maternal age, BMI before pregnancy, gestational weight gain, maternal education level, parity, infant gender, and region) were explored to elucidate the main characteristics of total and sn-2 FA profiles in Chinese mature milk and to explore the potential factors influencing their composition.

## Materials and methods

### Subjects

From June to October 2018, women with singleton pregnancies and no diabetes, hypertension, and other chronic diseases were recruited during their obstetrician visits in Beijing (Beijing Maternity Hospital affiliated to the Capital Medical University) and Danyang (Danyang People's Hospital), China (Characteristics between the two study sites were shown in Table A1). These subjects were considered eligible if their infants were delivered at full term and breastfed. For this study, we excluded mothers with the following criteria: birth weight of infants <2,500 g or > 4,000 g; with mental health disorders; unable to answer questions and poor postpartum mood; participated in any nutrition or drug intervention research; took hormones and antibiotics recently; and tobacco use.

### Sample size

A sample size of 64 subjects was estimated by the G^*^Power 3.1.9 software with a significance level of 5%, power of 80%, and expected effect size of 0.3, considering the correlation between total and sn-2 fatty acid of human mature milk and their correlated factors by correlation test. Evaluated by the G^*^Power 3.1.9 software, the statistical power was 83% with a sample size of 70, a significance level of 5%, and an expected effect size of 0.3.

### Ethical and legal considerations

This study was conducted according to the guidelines laid down in the Declaration of Helsinki. All the procedures involving human subjects were approved by the Chinese Clinical Trial Registry with the registration number ChiCTR1800018766 (http://www.chictr.org.cn/listbycreater.aspx). Written informed consent was obtained from all subjects.

### Collection of information

Data on height, weight (pre-conception weight and gestational weight gain), age, parity, delivery mode, and infant gender were obtained *via* a questionnaire during sample collection. Information such as the mothers' physical condition and lifestyle aspects were also included in the questionnaire.

### Human milk collection

On the day when mothers were scheduled for an obstetrical examination, 40–100 days post-delivery, they were asked to breastfeed at 6–8 am and then use an electric breast pump to empty the milk from one of their breasts at home. When they went to the hospital later (9–11 am), the milk from the breast that was previously emptied was collected by an electric breast pump with the help of sampling persons. The milk was mixed and poured into centrifuge tubes which were later wrapped with tin foil and stored at −80°C for further analysis.

### Lipid extraction, measurement of total and sn-2 FAs

Total lipids were extracted from human milk by a revised Mojonnier method (24). The extracted lipids were saponified and the FA methyl esters were obtained by FA methylation, and then analyzed by gas chromatography (GC). Sn-2 monoglyceride (MAG) was hydrolyzed from triglyceride (TAG) and then analyzed by GC following the method by Sahin et al. (25) (see Supplementary material 1, which demonstrates detailed experimental steps).

### Statistical analysis

The contents of each FA and sn-2 FA were expressed as mean ± SD and range (minimum~maximum). All the data were tested for normal distribution using SPSS (Version 20) before analysis. The Spearman correlations between differences in the contents of the FA/sn-2 FA vs. differences in characteristics of mothers and infants (age, infant gender, pre-conception BMI, gestational weight gain, delivery mode, parity, maternal education level, and sampling site) were calculated. The multiple regression model was adopted to estimate the importance of the differences in characteristics of mothers and infants in explaining the dissimilarities in FA/sn-2 FA. All the statistical analyses were performed in the R (Version 4.1.2), using “psych” (26), “reshape2” (27), “relaimpo” (28), and “packfor” (29) packages. Statistical significance was set at a *P* < 0.05. Plots were generated by the “ggplot2” (30) package in R.

## Results

Among the 100 screened healthy volunteers, 25 were excluded for not providing complete questionnaire information and five subjects withdrew from the study. A total of 70 mature milk samples were obtained from 70 mothers. The main characteristics of the participants are described in Table 1.

**Table 1:** Characteristics of mothers and infants included.

**Characteristics**	**Mean**	**Standard deviation**
Age (years)	29.61	3.98
Pre-conception BMI (kg/m^2^)	21.04	2.66
Gestational weight gain (kg)	13.43	3.94
	**Number**	**Frequency**
**Region**		
Beijing	34	48.57%
Danyang	36	51.43%
**Delivery mode**		
Natural	33	47.14%
Cesarean	37	52.86%
**Parity**		
1	52	74.29%
>1	18	25.71%
**Infant gender**		
Male	34	48.57%
Female	36	51.43%
**Maternal education level**		
Bachelor degree or above	39	55.71%
College degree	12	17.14%
High school degree or below	19	27.14%

### Fat content and total FAs composition

As displayed in Table 2, the total lipid content varied amongst subjects, ranging from 1.33 to 7.25 g/100 g (mean: 3.47±1.52 g/100 g). In total, 34 FAs were detected and only 23 FAs whose levels of more than 0.1% (total fatty acids) were shown in Table 2. C4:0, C6:0, C11:0, C13:0, C21:0, C22:0, C24:0, C14:1, C24:1, C22:2, and C20:5*n*-3 were also detected, but the levels were less than 0.1%. In general, monounsaturated fatty acid (MUFA) was the predominant FA (37.57 ± 3.82%), in which C18:ln-9c was found to make up the largest proportion (34.50 ± 3.44%). SFA was the second most abundant FA (34.50 ±3.44%), the largest component of which was C16:0 (20.06 ± 2.20%). The proportion of polyunsaturated fatty acids (PUFA) was the least (27.94±4.36%), among which C18:2n-6c accounted for more than 85% (24.08 ± 4.42%).

**Table 2:** Total lipid content (g/100 g milk) and fatty acid (% total fatty acids) levels in mature milk^**a**^.

**Lipids**	**All samples (*n* = 70)**	
	**Mean±SD**	**Range**
Total lipid g/100 g	3.47 ± 1.52	1.33~7.25
Total SFA, %	34.49 ± 3.88	25.98~44.53
C8:0	0.11 ± 0.05	0.03~0.22
C10:0	0.97 ± 0.33	0.29~1.77
C12:0	3.66 ± 1.43	0.77~7.28
C14:0	3.38 ± 1.34	1.32~7.34
C15:0	0.13 ± 0.05	0.07~0.34
C16:0	20.06 ± 2.20	14.76~25.14
C17:0	0.22 ± 0.04	0.15~0.43
C18:0	5.56 ± 1.01	3.77~8.28
C20:0	0.19 ± 0.08	0.10~0.45
Total MUFA, %	37.57 ± 3.82	29.15~49.43
C16:1	1.91 ± 0.46	1.03~3.18
C17:1	0.18 ± 0.03	0.09~0.23
C18:1n-9t	0.17 ± 0.20	0.01~1.45
C18:1n-9c	34.50 ± 3.44	26.63~44.33
C20:1	0.61 ± 0.30	0.27~1.89
C22:1n-9	0.20 ± 0.28	0.03~1.46
Total PUFA, %	27.94 ± 4.36	19.90~41.62
C18t^b^	0.61 ± 0.29	0.27~2.07
C18:2n-6c (LA)	24.08 ± 4.42	15.37~39.21
C18:3n-6	0.14 ± 0.06	0.05~0.27
C20:2	0.45 ± 0.12	0.29~1.17
C20:3n-6	0.40 ± 0.14	0.18~0.88
C20:4n-6 (ARA)	0.57 ± 0.14	0.32~1.17
Total n-6PUFA	25.70 ± 4.42	17.04~40.5
C18:3n-3 (ALA)	1.68 ± 0.73	0.47~3.29
C22:6n-3 (DHA)	0.31 ± 0.15	0.11~0.96
Total n-3PUFA, %	2.05 ± 0.76	0.86~3.64
n-6PUFA/n-3PUFA	14.60 ± 6.69	5.46~36.15
LA/ALA	17.89 ± 9.67	5.87~42.11
ARA/DHA	2.14 ± 0.76	0.72~4.17

^a^The fatty acids with average content > 0.1% are shown in the table.

^b^C18t is the sum of C18:1t, C18:2t and C18:3t, representing the level of trans FAs.

LA, linoleic acid; ARA, arachidonic acid; ALA, α-linolenic acid; DHA, Docosahexaenoic acid.

### Sociodemographic determinants of total FAs

**Figure 1:**
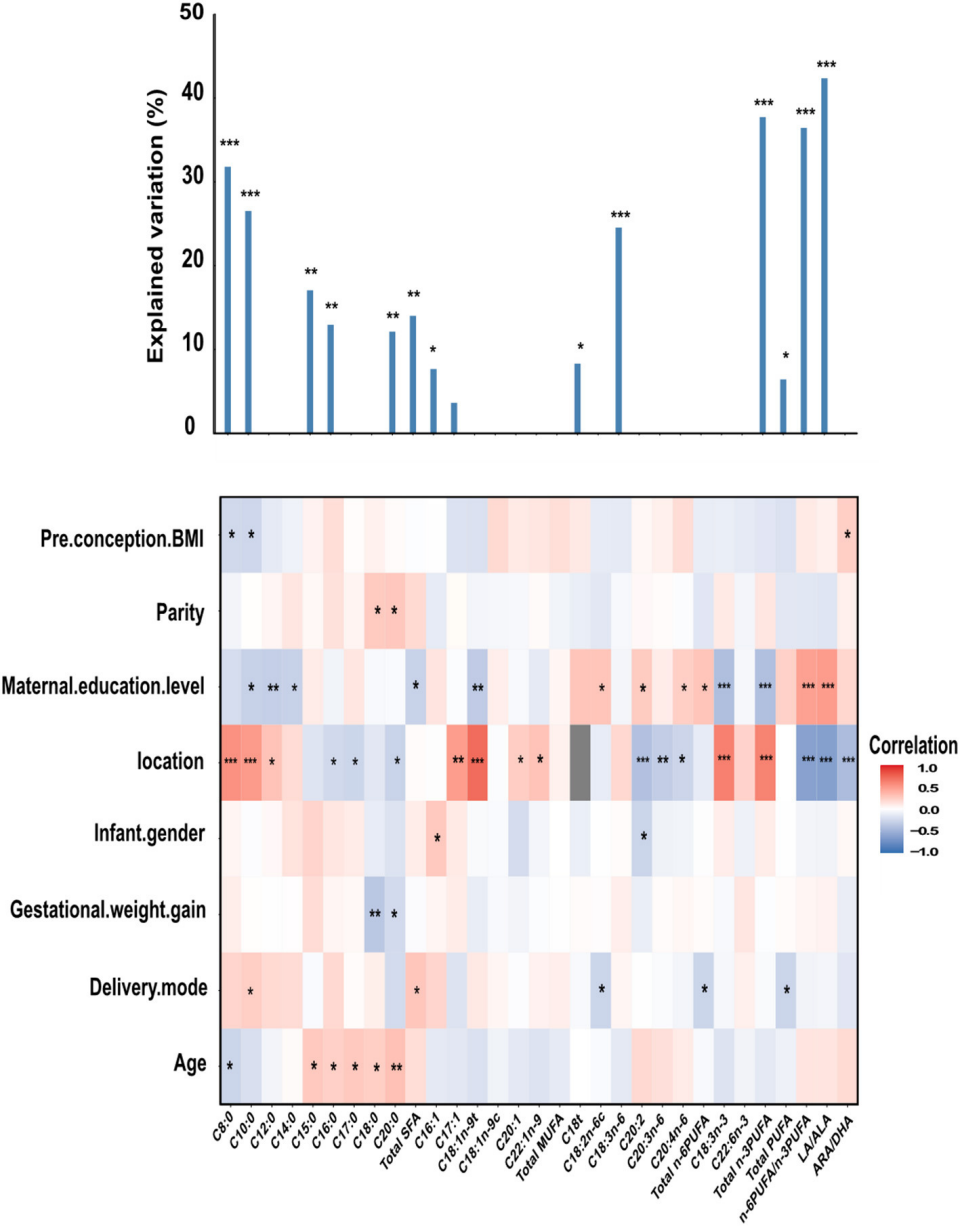
The column chart shows the total contribution of indicators to the interpretation of FAs variation (obtained by multiple linear regressions). The heat map shows the Spearman correlations between total FAs and correlated factors. Coloring reflects the direction and magnitude of the correlation coefficients. For pre-conception BMI, parity, maternal education level, gestational weight gain, and age, the higher the value/level of the correlated factors, the higher the fatty acid content. For the location, red indicates FAs in Danyang are higher than that in Beijing. For infant gender, red indicates FAs in mothers with baby girls are higher than that with baby boys. For delivery mode, red indicates FAs of mothers with natural childbirth are lower than with other modes of delivery. The major predictors were identified based on the correlation and best multiple regression model. C18t is the sum of C18:1t, C18:2t, and C18:3t, representing the level of trans FAs. LA, linoleic acid (C18:2n-6c); ARA, arachidonic acid (C20:4n-6); ALA, α-linolenic acid (C18:3n-3); DHA, Docosahexaenoic acid (C22:6n-3). *P < 0.05; **P < 0.01; ***P < 0.001.

Some potential correlated factors of human milk FAs, namely, pre-conception BMI, parity, maternal education level, location, infant gender, gestational weight gain, delivery mode, and maternal age were individually explored using the Spearman correlation analysis and later tested using the multiple regression model (Figure 1).

A significant correlation was found between maternal education level and FAs (C10:0, C12:0, C14:0, Total SFA, C18:1n-9t, C18:2n-6c, C20:2, C20:4n-6, Total n-6PUFA, C18:3n-3, Total n-3PUFA, n-6PUFA/n-3PUFA, and LA/ALA), and location and FAs (C8:0, C10:0, C12:0, C16:0, C17:0, C20:0, Total SFA, C17:1, C18:1n-9t, C20:1, C22:1n-9, C20:2, C20:3n-6, C20:4n-6, C18:3n-3, Total n-3PUFA, n-6PUFA/n-3PUFA, and LA/ALA). The milk of mothers from Danyang had higher levels of C8:0, C10:0, C12:0, C17:1, C18:1n-9t, C20:1, C22:1n-9, C18:3n-3, and total n-3PUFA, and lower levels of C16:0, C17:0, C20:0, C20:2, C20:3n-6, C20:4n-6 and lower n-6PUFA/n-3PUFA, LA/ALA, and ARA/DHA ratios. Compared to the milk of mothers with a high school degree or below, those with a bachelor's degree or above had lower C10:0, C12:0, C14:0, Total SFA, C18:1n-9t, C18:3n-3, Total n-3PUFA, and higher C18:2n-6c, C20:2, C20:4n-6, Total n-6PUFA, and n-6PUFA/n-3PUFA, and LA/ALA ratios.

Several associations were observed between age and some SFAs (C8:0, C15:0, C16:0, C17:0, C18:0, and C20:0), delivery mode, and C10:0, Total SFA, C18:2n-6C, Total n-6PUFA, and Total PUFA. For pre-conception BMI, parity, infant gender, and gestational weight gain, there are few correlations shown between these characteristics and FAs.

For C8:0, LA/ALA, n-6PUFA/ n-3PUFA, and total n-3PUFA, these factors can explain more than 30% of the variance in mature milk FAs.

### The composition of sn-2 FAs

In this study, 26 sn-2 FAs were examined. In total, 8 major sn-2 FAs were detected in mature breast milk (Table 3), namely, C16:0, C18:2n-6, C18:1, C14:0, C12:0, C18:0, C16:1, and C18:3n-3. These FAs collectively accounted for 92.43% (mean) of sn-2 FAs. Different from the total FAs composition, total SFA (mean: 67.74 ± 5.21%) was predominant in the sn-2 position, followed by MUFA (16.26 ± 2.54%) and PUFA (16.00 ± 3.87%). Notably, C16:0 accounted for more than half of the total sn-2 FAs (50.18 ± 4.74%). Furthermore, approximately 80% of the total C16:0 was in the sn-2 position (79.72 ± 4.29%). C14:0 and C15:0 were also mainly found in the sn-2 position; the average relative molar percentages at the sn-2 position were 66.80 and 75.31%, respectively. However, most MUFAs and PUFAs were located at sn-1, 3 positions.

**Table 3:** Composition of sn-2 fatty acids and relative molar percentage^a^ of each fatty acid in mature milk at the sn-2 position (%).

**Lipids**	**sn-2 fatty acids**		**Relative molar percentage**	
	**Mean±SD**	**Range**	**Mean±SD**	**Range**
C8:0	0.08 ± 0.03	0.02~0.15	23.86 ± 11.13	11.10~61.67
C10:0	0.80 ± 0.28	0.22~1.75	27.66 ± 9.85	6.90~64.87
C12:0	5.23 ± 1.96	1.18~12.38	47.23 ± 9.49	10.89~68.26
C14:0	6.83 ± 2.46	2.95~15.05	66.80 ± 10.54	18.98~90.55
C15:0	0.31 ± 0.09	0.18~0.66	75.31 ± 11.04	45.11~95.24
C16:0	50.18 ± 4.74	36.03~61.42	79.72 ± 4.29	64.29~86.82
C17:0	0.26 ± 0.06	0.15~0.54	39.12 ± 6.51	23.92~58.94
C18:0	3.79 ± 1.25	1.78~7.60	22.35 ± 7.96	10.15~47.51
C20:0	0.22 ± 0.09	0.11~0.54	39.05 ± 13.79	15.10~77.78
C22:0	0.05 ± 0.04	0.00~0.17	24.55 ± 17.67	0.31~80.45
C14:1	0.11 ± 0.17	0.02~0.75	29.36 ± 12.82	10.26~72.67
C16:1	2.72 ± 0.61	1.18~4.48	46.56 ± 9.39	26.15~68.30
C17:1	0.18 ± 0.05	0.06~0.33	29.91 ± 9.56	11.02~57.09
C18:1t	0.12 ± 0.10	0.04~0.75	32.54 ± 23.49	9.81~87.57
C18:1	12.78 ± 2.26	7.93~22.43	11.86 ± 1.69	8.50~17.14
C20:1n-9	0.30 ± 0.14	0.11~0.92	16.34 ± 3.60	8.87~30.25
C22:1n-9	0.17 ± 0.21	0.02~0.91	25.84 ± 8.54	12.56~49.40
C18:2t	0.18 ± 0.11	0.06~0.84	21.21 ± 5.41	12.85~42.30
C18:2n-6	13.49 ± 3.61	7.64~29.91	17.85 ± 2.55	12.17~24.57
C18:3n-6	0.12 ± 0.06	0.03~0.45	27.48 ± 12.11	11.13~83.74
C18:3t	0.16 ± 0.11	0.05~0.78	34.89 ± 18.23	15.10~81.34
C20:2n-6	0.18 ± 0.05	0.05~0.31	12.48 ± 2.83	3.39~18.04
C20:3n-6	0.14 ± 0.18	0.04~1.09	8.02 ± 4.39	3.85~25.73
C20:4n-6	0.30 ± 0.09	0.11~0.56	17.23 ± 4.11	7.88~25.74
C18:3n-3	1.20 ± 0.53	0.26~2.82	23.31 ± 5.43	12.83~49.81
C22:6n-3	0.33 ± 0.12	0.04~0.62	37.79 ± 15.57	6.68~80.62

^a^calculated as (M/T/3) ×100, M, molar percentage of sn-2 fatty acid; T, molar percentage of total fatty acid.

### Sociodemographic determinants of sn-2 FAs

**Figure 2:**
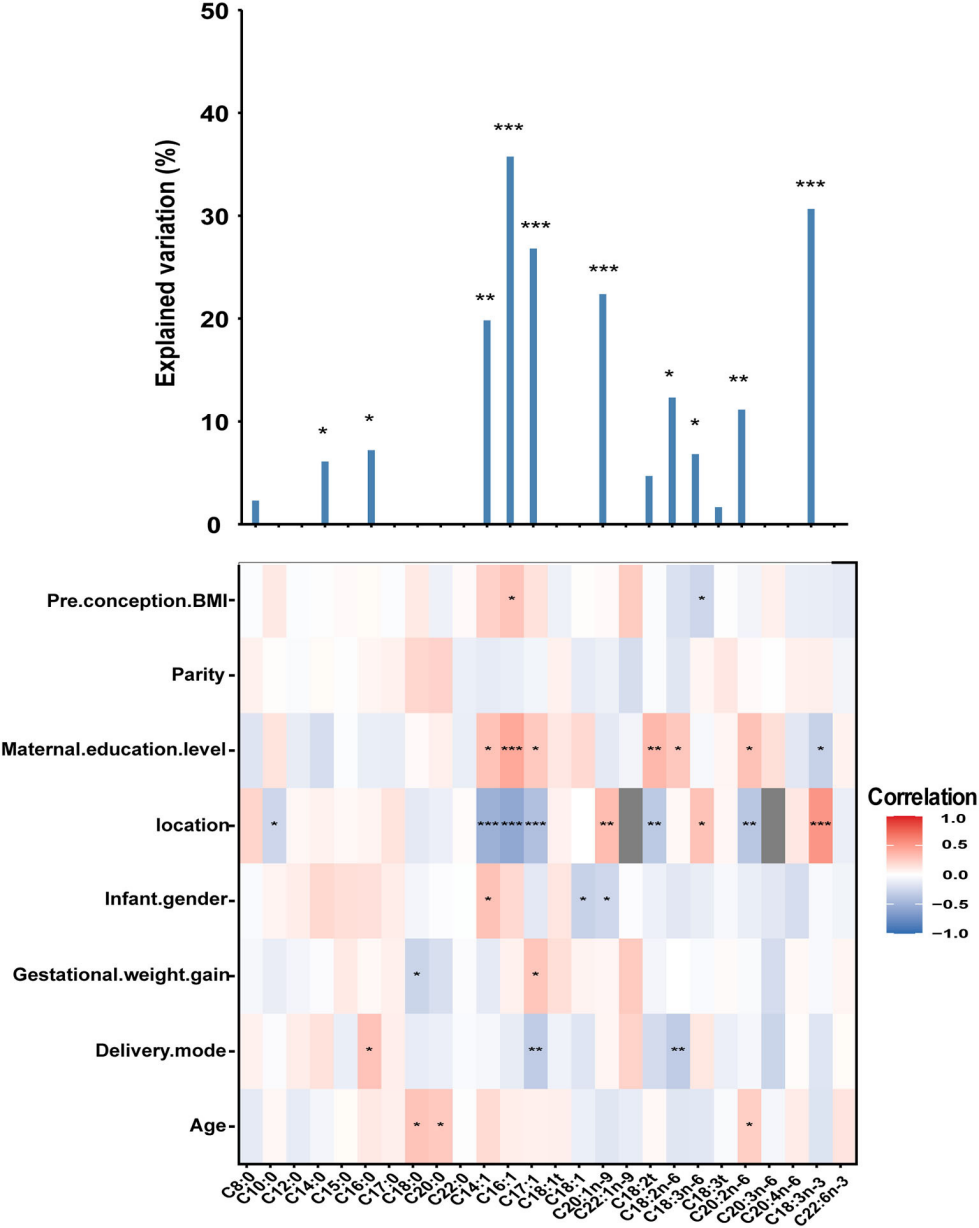
The column chart shows the total contribution of indicators to the interpretation of sn-2 FAs variation (obtained by multiple linear regressions). The heat map shows the Spearman correlations between sn-2 FAs and correlated factors. Coloring reflects the direction and magnitude of the correlation coefficients. For pre-conception BMI, parity, maternal education level, gestational weight gain, and age, the higher the value/level of the correlated factors, the higher the fatty acid content. For the location, red indicates FAs in Danyang are higher than that in Beijing. For infant gender, red indicates FAs in mothers with baby girls are higher than that with baby boys. For delivery mode, red indicates FAs of mothers with natural childbirth are lower than with other modes of delivery. The major predictors were identified based on correlation and the best multiple regression model. DHA, Docosahexaenoic acid (C22:6n-3). *P < 0.05; **P < 0.01; ***, *P* < 0.001.

As shown in Figure 2, no associations with statistical significance were observed between sn-2 FAs and parity. Similar to FAs, maternal education level and location were strong predictors for differences in the constitution of mature milk sn-2 FAs. Mothers from Beijing had lower C20:1n-9, C18:3n-3, and C18:3n-6 and higher percentages of C10:0, C14:1, C16:1, C17:1, C18:2t, and C20:2n-6. Mothers with a bachelor's degree or above had higher C14:1, C16:1, C17:1, C18:2t, C18:2n-6, C20:2n-6, and lower C18:3n-3 compared to the mothers with a high school degree or below. Several correlations were also found between age and C18:0, C20:0, and C20:2n-6; delivery mode and C16:0, C17:1 and C18:2n-6; gestational weight gain and C18:0, C17:1; infant gender and C14:1, C18:1, C20:1n-9; pre-conception BMI and C16:1, C18:3n-6.

These factors explained 1.67–35.76% of the variance in sn-2 FAs, with the lowest proportion (1.67%) for C18:3t and the highest proportion (35.76%) for C16:1.

## Discussion and conclusions

In this present study, we analyzed the total and sn-2 FA profiles in mature milk samples from healthy lactating women in Beijing and Danyang, China. We found specific associations of pre-conception BMI, maternal education level, location, infant gender, gestational weight gain, delivery mode, and maternal age with total and sn-2 FAs in mature milk. Parity may affect total FA composition but not sn-2 FA. Location and maternal education level were strong predictors for differences in the constitution of mature milk total and sn-2 FAs.

### Fat content and total FAs

Total lipid content in mature breast milk found in our study was 3.47 ± 1.52 g/100 g, which was close to the level (3.39 ± 1.24 g/dl) found in a recent systematic review in Chinese women (31). Our study showed that the total contents of SFA, MUFA, and PUFA in mature milk were 34.49 ± 3.88%, 37.57 ± 3.82%, and 27.94 ± 4.36%, respectively, which was similar to the pooled results of the Asian populations in a meta-analysis study (37.87~39.91%, 34.64~36.72%, 23.18~29.79% for SFA, MUFA, PUFA, respectively) (32). It is worth noting that the infant milk formula standards of China (GB10765-2010) specify that the ratio of LA/ALA should range from 5:1 to 15:1, which is in line with the standards of international organizations and other countries, e.g., the Food and Agriculture Organization of the United Nations, WHO, Australia and New Zealand (33). However, the mean value for LA:ALA in our study was 17.89:1; other studies conducted among the Chinese population also showed a mean value for LA/ALA in mature milk above 10:1 [Shanghai:18.56:1 (34); Guangzhou:19.70:1 (35); Beijing:15.69:1 (35), Jiangsu:11.9:1 (35)]. It is suggested that we should focus on the characteristics of domestic breast milk to guide the formulation and production of infant formula, rather than just referring to standards that are based on data from the breast milk of mothers in western countries. In our study, the sampling site had a significant effect on PUFAs. The levels of n-6 series of long-chain polyunsaturated fatty acids (LCPUFA) (C20:2, C20:3n-6, C20:4n-6, n-6PUFA/n-3PUFA, LA/ALA, and ARA/DHA) in mature milk from mothers in Beijing were higher than that of Danyang, while the opposite was observed in the n-3 series of LCPUFA (C18:3n-3 and Total n-3PUFA). The discrepancy in eating habits between the two regions might lead to the difference in the proportions of PUFA in the mature milk. Beijing is an inland city, while Danyang is a coastal city abundant in various fishes rich in n-3 PUFA. In addition to PUFA, regional differences were also observed in several types of SFA (C8:0, C10:0, C12:0, C16:0, C17:0, and C20:0) and MUFA (C17:1, C18:1n-9t, C20:1, and C22:1n-9), which might be caused by other site-specific correlated factors, such as gene, climate, or lifestyle.

Maternal education level was also found to be associated with some human milk FAs. Mothers with higher degrees had lower SFAs (C10:0, C12:0, C14:0, and Total SFA), n-3 series of LCPUFA (C18:3n-3 and Total n-3PUFA), C18:1n-9t, and higher n-6 series of LCPUFA (C18:2n-6c, C20:2, C20:4n-6, Total n-6PUFA, and n-6PUFA/n-3PUFA, LA/ALA ratios). In a similar manner, a study on low-income Indian women showed that a higher maternal education resulted in lower concentrations of SFAs and PUFAs (36). However, Al-Tamer and Mahmood (37) found the proportions of the n-3 series of LCPUFA decreased with decreasing socioeconomic status (mother's education and occupation). The effect of maternal education on the FA in human milk is equivocal. A lower level of education usually implies higher unemployment or lower wages and a lower income level (38). Besides, as a reflection of traditional gender roles in society, women are more likely to take on the responsibility of food selection and acquisition. Taking all into consideration, maternal education level may influence the nutritional knowledge and income levels, which may lead to the difference in food consumption and dietary habits, and therefore, affect the profiles of FAs.

The influence of maternal age on FAs is ambiguous. In this study, maternal age is positively associated with some SFAs (C15:0, C16:0, C17:0, C18:0, and C20:0). Antonakou et al. (39) also reported that maternal age was an independent factor of MUFAs. However, two studies (40, 41) reported that maternal age was not related to milk lipids. Moreover, the association between infant gender and FAs was observed in our study. Infant gender was reported to influence hormonal secretions in the placenta during pregnancy, which is related to breast development (42). This may help explain the associations found in our study.

The results also showed that the mature milk of mothers who had delivered with a natural birth contained fewer SFAs (C10:0 and Total SFA) and more PUFAs (C18:2n-6c, Total n-6PUFA, and Total PUFA) compared with the cesarean section group. Sinanoglou et al. investigated the factors affecting human colostrum FAs and found that the proportions of C12:0, C14:0, C18:3n-3, C20:4n-6, C20:5n-3, and Total n-3PUFA were significantly (*P* < 0.05) lower in colostrum fat from cesarean than from vaginal deliveries (43). Gestational weight gain, infant gender, parity, and pre-conception BMI were also found to be associated with several human milk FAs, suggesting a possible role between these characteristics in FAs. As few studies have examined the associations between maternal characteristics and FAs, more research is needed to clarify these factors.

### Sn-2 FAs

The FAs at the sn-2 position of TAG in human milk were reported to significantly affect the absorption of FAs and calcium, infant intestinal flora (17), and stool consistency (44). TAGs with sn-2 FAs have recently become a target in the optimizing of infant formula (45–47).

Results of our study corresponded with Deng et al. (22), in which several SFAs (C14:0, C15:0, and C16:0) were mainly acylated in the sn-2 position, and most MUFAs and PUFAs showed sn-1,3 positional selectivity in TAGs.

Notably, the sn-2 FA profile seemed to be less affected by the factors that influence total FA. Parity was not found to be associated with sn-2 FAs. The association between location and several FAs (C20:1n-9, C18:3n-3, and C20:2n-6) in total FAs was similar to that in sn-2 FAs. The sn-2 FAs as a part of total FAs may account for the similarity regarding these correlations between total and sn-2 FAs. C16:0 and DHA are proved to be beneficial to infants' health under the sn-2 positional selectivity (17, 44, 48). However, DHA was not observed to be associated with the factors discussed earlier, and C16:0 was only found to be associated with the delivery mode. This may suggest that the levels of the two FAs are relatively constant. The average levels of C16:0 and DHA at the sn-2 position among the domestic population may be a reliable reference for their infant milk powder formulation.

Based investigation of the profiles of FAs and sn-2 FAs in human mature milk samples from two representative areas in China, this study also strived to explore the associations between maternal factors and FAs, especially, sn-2 FAs, which are rarely explored in previous studies. Our study has a few limitations. First, a systematic dietary survey was not conducted, so nutrient intakes cannot be estimated, and the effects of maternal diet on FAs cannot be explored. In addition, we restricted our analyses to several sociodemographic factors, but other elements, namely, lactation stage, genes, and gestational age, were also reported to influence the FA profile. Further research is needed to integrate all the correlated factors. Despite these limitations, one of the outstanding advantages of this study is that the sampling sites were restricted to the hospital, and breast pump sampling was used to reduce the confounding factors of sampling, which significantly improved the reliability of the results.

In conclusion, this study elucidated the total and sn-2 FA profiles of mature milk in women from Beijing and Danyang, China. Correlation analysis revealed that the total FAs composition was variable and independently associated with location, maternal age, infant gender, pre-conception BMI, gestational weight gain, delivery mode, parity, and maternal education level. On the contrary, sn-2 FAs composition seemed more constant than total FAs, as parity was not found to be associated with the levels of sn-2 FAs. The conspicuous contribution of location and maternal education level was observed in both total and sn-2 FAs, which implicated the possible role of economic–related or education–related dietary habits; the delivery mode was also a significantly correlated predictor of the variation in FAs and sn-2 FAs. Together, these findings present a pilot study on the correlated factors of FAs in mature milk and may act as a reference for infant formula or human milk fortifier optimization.

## Data availability statement

The raw data supporting the conclusions of this article will be made available by the authors, without undue reservation.

## Ethics statement

The studies involving human participants were reviewed and approved by Chinese Clinical Trial. The patients/participants provided their written informed consent to participate in this study.

## Author contributions

MN conducted the experiments, analyzed the data, and wrote the first draft of the manuscript. YW contributed to the conception and design of the study. ZY wrote a part of the first draft. XX and HZ provided experimental and technical support. JC contributed to manuscript revision, read, and approved the submitted version. YY and LZ supervised. All the authors contributed to the article and approved the submitted version.

## Funding

This study was funded by the Yihai Kerry Group.

## Conflict of interest

Authors XX and HZ were the employees of the Wilmar (Shanghai) Biotechnology Research & Development Center Co., Ltd. when this work was done. They participated in the validation of the study and the opinions they expressed were their own and do not necessarily reflect the views or recommendations of their respective affiliations. The remaining authors declare that the research was conducted in the absence of any commercial or financial relationships that could be construed as a potential conflict of interest.

## Publisher's note

All claims expressed in this article are solely those of the authors and do not necessarily represent those of their affiliated organizations, or those of the publisher, the editors and the reviewers. Any product that may be evaluated in this article, or claim that may be made by its manufacturer, is not guaranteed or endorsed by the publisher.
