# Supplementary material for: Profiles of total and sn-2 fatty acid of human mature milk and their correlated factors: A cross-sectional study in China
Source: Front Nutr. 2022 Aug 22;9:926429. doi: 10.3389/fnut.2022.926429 (PMC9441907; doi:10.3389/fnut.2022.926429)
Supplement: Supplementary file 1 [file Table_1.DOCX]

Table A1. Available characteristics between the two study sites*

| Site | Beijing | Jiangsu (Dangyang) |
| --- | --- | --- |
| Longitude and latitude | 115.7 °-117.4 ° E, 39.4 ° - 41.6 ° N | 119 ° 23 ′～ 119 ° 53 ′ E and 31 ° 44 ′～ 32 ° 08 ′ N |
| Total Area (Km^2^) | 16410 | 1047 |
| Climate | Warm temperate semi humid and semi-arid monsoon climate | Subtropical monsoon climate |
| City type | Inland city | Coastal city |
| Resident population (2020) | 21893000 | 988900 |
| Regional GDP (billion RMB, 2020) | 3610.26 | 118 |
| Per capita disposable income of residents (RMB, 2020) | 69434 | 54000 (Urban) |

*Data from Wikipedi
