# Supplementary material for: Profiles of total and sn-2 fatty acid of human mature milk and their correlated factors: A cross-sectional study in China
Source: Front Nutr. 2022 Aug 22;9:926429. doi: 10.3389/fnut.2022.926429 (PMC9441907; doi:10.3389/fnut.2022.926429)
Supplement: Supplementary file 2 [file Data_Sheet_1.docx]

**Fat extraction**

The steps were as follows: The corresponding volume of ammonia water was added in the ratio of 5:1 (sample/ammonia water, v/v) to the liposuction bottle containing homogeneous sample. The mixture was incubated in a water bath (65℃) for 15 minutes. Then ethanol was added to the liposuction bottle in the ratio of 1:1 (sample: ethanol, v/v) when the liposuction bottle was cooled to room temperature. After full mixing, anhydrous ether was added in the ratio of 1:2.5 (sample: anhydrous ether, v/v). Then the same volume of petroleum ether as anhydrous ether was added, the mixture was extracted again as described above. After centrifugation, the upper liquid was poured into the fat collection bottle. The first addition of 1/2 ethanol was added to the liposuction bottle for a second extraction. The above procedure was repeated for 1-2 times with anhydrous ether and petroleum ether.

**FAs composition**

The conical flask containing the fat extract and sodium hydroxide methanol solution（2M） was connected to a reflux condenser, refluxed for 20 min in a water bath at 80 ° C ± 1 ° C, 7 ml of boron trifluoride was added from the upper end of the reflux condenser, and reflux was continued for 3 min. The reflux condenser was rinsed with 2 ml of methanol solution, the flask was removed from the water bath, and rapidly cooled to room temperature. 5 ml of n-heptane was added, and the mixture was shaken for 3 minutes. Saturated NaCl solution (2ml) was added and the layer was allowed to stand for separation. The supernatant was puriﬁed through a 0.22µm needle ﬁlter and analyzed by gas chromatography (GC).

**Sn-2 FAs composition**

Lipase (Pancreatic lipase L3126-100G, 20 mg), Tris buffer solution (pH 8.0, 2 mL), sodium cholate solution (0.1%, 0.5mL) and calcium chloride solution (22%, 0.2 mL) were added to the centrifugal tube containing about 0.1g HM fat. The tube was put into a 40℃ water bath immediately for 1 minute with continuously shaking. Then hydrochloric acid (6M, 1 mL) and ether (1 mL) was added immediately to the tube after shaking violently for 2 minutes with oscillator. The centrifugal tube was put into the centrifuge (1792 RCF, 6 minutes). The organic phase was transferred to the clean test tube and was evaporated to one third of the original volume by nitrogen gas. The 2-monoglyceride in organic phase was separated by Thin Layer Chromatography (TLC), and the developing solvents were petroleum ether /anhydrous ether/formic acid (70:30:1, v/v/v). The silica gel plate was placed in an ultraviolet observation chamber (wavelength 254 nm). The band of 2-monoglyceride was circled out and scraped off. The FA methyl ester was prepared by methyl esterification reaction and analyzed by GC.

GC analysis conditions: a) capillary column: 30m*0.32m*0.25μm, HP-5 (Agilent, California, USA); b) injector temperature: 270℃; c) detector temperature: 280℃; d) programmed temperature: initial temperature was 100℃ for 13 minutes, then raised to 180℃ at the rate of 10℃/min and maintained for 6 minutes, followed by an increase to 200℃ at the rate of 1℃/min and held at 200℃ for 6 minutes. At last, the temperature rised to 280℃ at the rate of 4℃/min and lasted for 10 minutes.; e) carrier gas: nitrogen; f) shunt ratio: 100:1; g) injection volume: 1.0 μL.
